# The collateral circulation of the heart

**DOI:** 10.1186/1741-7015-11-143

**Published:** 2013-06-04

**Authors:** Pascal Meier, Stephan H Schirmer, Alexandra J Lansky, Adam Timmis, Bertram Pitt, Christian Seiler

**Affiliations:** 1 The Heart Hospital London, University College London Hospitals UCLH, London, UK; 2 Division of Cardiology, Yale Medical School, New Haven, CT, USA; 3 Department of Cardiology, University Hospital Saarland, Saarland, Germany; 4 NIHR Biomedical Research Unit, Barts and the London School of Medicine and Dentistry, London Chest Hospital, London, UK; 5 Division of Cardiology, University of Michigan Medical Center, Ann Arbor, MI, USA; 6 Department of Cardiology, University Hospital Bern, Bern, Switzerland

**Keywords:** Angiogenesis, Arteriogenesis, Coronary artery disease, Coronary collateral circulation

## Abstract

The coronary arteries have been regarded as end arteries for decades. However, there are functionally relevant anastomotic vessels, known as collateral arteries, which interconnect epicardial coronary arteries. These vessels provide an alternative source of blood supply to the myocardium in cases of occlusive coronary artery disease. The relevance of these collateral arteries is a matter of ongoing debate, but increasing evidence indicates a relevant protective role in patients with coronary artery disease. The collateral circulation can be assessed by different methods; the gold standard involves intracoronary pressure measurements. While the first clinical trials to therapeutically induce growth of collateral arteries have been unavailing, recent pilot studies using external counterpulsation or growth factors such as granulocyte colony stimulating factor (G-CSF) have shown promising results.

## Introduction

**Figure 1:**
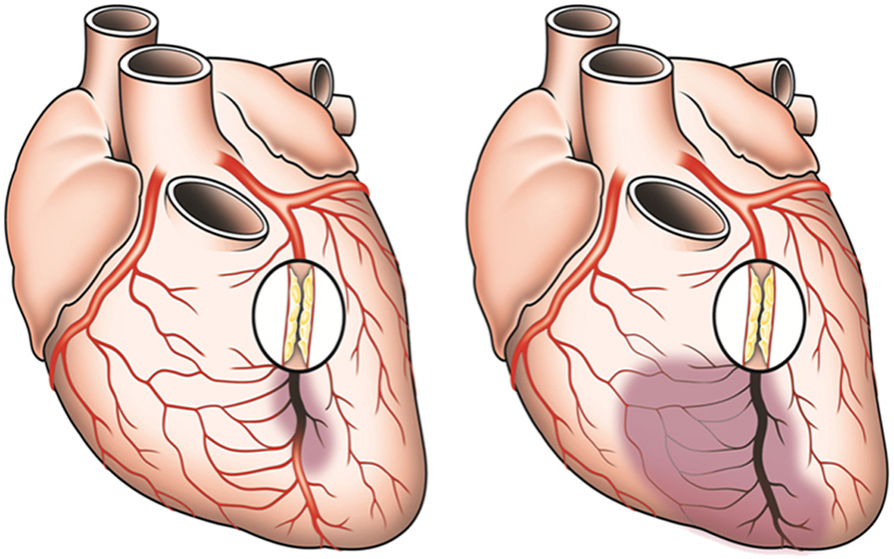
**Schematic drawing of the coronary artery circulation with (left panel) and without (right panel) interarterial anastomoses between the right coronary artery and the occluded left anterior descending artery (LAD; occluded beyond the third diagonal branch).** The gray area indicates the area at risk for myocardial infarction in case of the LAD occlusion and in the absence of collaterals (corresponding to the infarct size in the example on the right side). (Illustration by Anne Wadmore, Medical Illustrations Ltd, London, UK).

Anastomotic channels, known as collateral vessels, connect a territory supplied by one epicardial coronary artery with that supplied by another [1]. Collateral arteries therefore provide an alternative source of blood supply to myocardium that has been jeopardized by occlusive coronary artery disease, and they can help to preserve myocardial function in the setting of coronary artery disease [2] (Figure 1).

While their growth is often thought to be initiated by ischemia, collateral arteries are also present in individuals who do not have coronary disease [2,3]. Obviously, other factors seem to play a more important role.

Although collateral blood flow after epicardial coronary occlusion may be sufficient in some patients to meet myocardial needs at rest, the prevalent view is that collateral circulation is generally not sufficient to meet myocardial demands during exercise [4] and may not prevent myocardial ischemia during coronary occlusion. To prevent myocardial ischemia during acute vessel occlusion, a flow of 20% to 25% is generally regarded as sufficient to provide the blood supply needed at rest [5]. One in four patients without coronary artery disease has sufficient collaterals as compared with one in three patients with coronary artery disease [3,6]. The reasons for this are not fully understood, but genetic factors are likely to play a role [7-9].

### Assessing the collateral circulation

How can collateral function be measured? Except for the situation with a known chronic total coronary occlusion, there is currently no technique to quantify the collateral circulation non-invasively in human. The easiest strategy is the visual assessment of collateral arteries by coronary angiography. This can be performed in a semiquantitative manner as described by Rentrop *et al*. [10]. The Rentrop method involves balloon occlusion of the contralateral coronary artery, which is rarely performed. Collateral vessels from patent to occluded are classified ranging from grade 0 (no visible filling of any collateral channel), grade 1 (filling of the side branches of the occluded artery, with no dye reaching the epicardial segment), grade 2 (partial filling of the epicardial vessel), and grade 3 (complete filling of the epicardial vessel by collaterals) [11].

Instead, most clinicians and investigators apply the Rentrop score without occluding the contralateral vessels. However, a patent contralateral coronary artery increases the back pressure in this collateral-receiving territory, which underestimates the degree of collateralization. This visual method has several other limitations: it is not a very objective measure, and it is influenced by blood pressure and the force of contrast injection as well as the duration of filming.

The currently most accurate assessment method measures the so-called collateral flow index (CFI). Two methods are available: one is based on Doppler velocity measurements, which is limited by frequent artifacts. The second one is more accurate and based on pressure measurement. For the Doppler approach, the collateralization of a certain coronary artery can be measured by placement of a Doppler sensor tipped guide wire. Then, the antegrade flow through the coronary artery needs to be blocked with an angioplasty balloon. The flow velocity measured with the Doppler sensor distal to the occluded vessel derives from collaterals. Then, the vessel is angioplastied so that there is no remaining lesion and the flow velocity measured again, which represents the flow through the normal vessel. The collateral flow velocity is then compared to the flow velocity through the open coronary artery and indicates the percentage of normal blood flow that can be preserved via the collateral circulation in case of an abrupt vessel occlusion [12].

### Pressure-based CFI

The pressure index of the distal pressure during vessel occlusion divided by the systemic blood pressure both subtracted by central venous pressure measures a pressure-derived CFI. The central venous pressure has to be taken into account as a back pressure [2]. Another, simpler, cheaper and very accurate way to measure collateral function is an intracoronary electrocardiogram (ECG) [5]. Studies have defined a threshold of ST-segment elevation ≥0.1 mV during a 1 to 2 minute vessel occlusion with an angioplasty balloon to define insufficient collateralization. In addition, if the patient develops chest pain during balloon occlusion of the vessel, this can be regarded as a sign of insufficient collateral function.

All three methods, Rentrop score, CFI and intracranial ECG, predict clinical outcomes and are therefore useful [5,13]. For research purposes, the CFI is clearly superior because it is a continuous value while the Rentrop score is an ordinal and the ECG a dichotomous variable. The CFI is therefore more informative and increases the statistical power.

Other methods have been described, such as ‘wash-out collaterometry’ whereby the time to contrast dye clearance distal to a balloon occluded artery is measured. The washout is quicker the better the vessel is collateralized [14]. However, in contrast to the above-mentioned methods, none of these have shown a predictive value in clinical practice.

### Determinants of the collateral circulation

The degree of collateralization varies considerably among patients. For many years, ischemia has been believed to be the underlying stimulus for collateral growth. However, no study could directly prove a causative role for ischemia in the induction of collateral growth.

Clinical studies have described several independent clinical and angiographic variables that correlate with the degree of collateralization. In healthy individuals, these include hypertension and resting heart rate [15], while variables in patients with coronary artery disease include severity of coronary stenosis [6,16], longer duration of angina [16], proximal lesion location [16], and longer duration of lesion occlusion [17] (Table 1).

**Table 1:** Clinical factors that can influence collaterals

**Factor (reference)**	**Remarks**
Degree of coronary stenosis [16]	Strongest predictor, confirmed in several studies
Proximal lesion location [16]	
Longer duration of symptoms [16]	
Longer duration of lesion occlusion [17]	In patients with chronic total occlusions
Heart rate (lower) [15]	Only in patients without coronary artery disease

### Mechanism of collateral growth (arteriogenesis)

**Figure 2:**
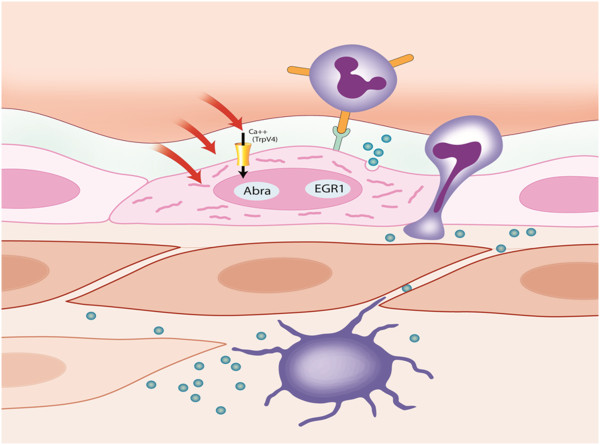
**Mechanism of induction of collateral growth (arteriogenesis).** (1) Endothelium senses shear stress via Ca+ channels, transduction via glycocalyx and cytoskeleton. (2) Actin-binding Rho-activating protein (ABRA) and early growth response protein 1 (EDGR1) genes are upregulated. (3) Activated endothelium expresses adhesion molecules such as intercellular adhesion molecule (ICAM) and growth factors such as monocyte chemoattractant protein 1 (MCP1) as well as NO. (4) Circulating monocytes bind their macrophage 1 antigen (Mac-1) receptors to ICAM. (5) Monocytes differentiate into macrophages and secrete additional growth factors and chemoattractants, stimulating proliferation of smooth muscle and endothelial cells. (Illustration by Anne Wadmore, Medical Illustrations Ltd).

The most important trigger for collateral growth, called arteriogenesis, is tangential fluid shear stress at the endothelial level along with recruitment of bone marrow derived mononuclear cells [2,18-20] (Figure 2). Following obstruction or occlusion of a major artery, a steep pressure gradient develops across the pre-existing collateral anastomoses. This pressure gradient is the driving force for an increase in blood flow through the collateral arterioles, leading to an augmented fluid shear stress that, in turn, activates the collateral arteriolar endothelium. The exact way by which the collateral endothelial cell senses the shear stress is still unrevealed. ‘Mechanosensation’ is a multifactorial process, and it is currently accepted that not only certain mechanosensitive channels on the endothelial surface are needed to convert the physical force into a cellular response, but that rather the cell as a whole, including its cytoskeleton [21], and the endothelial glycocalyx [22] acts as a mechanosensor [23]. There are, however, certain cation channels on the cell surface that are regarded to be direct receptors for mechanical forces (for example, shear stress: activated endothelium can, in turn, further set off the process of arteriogenesis). Cell adhesion molecules (intercellular adhesion molecule 1 (ICAM1), vascular cell adhesion molecule 1 (VCAM1)) are upregulated to facilitate adhesion of circulating mononuclear cells. Crosstalk with adjacent smooth muscle cells leads to the production of nitric oxide (NO) and other pro-arteriogenic molecules. Apart from tangential fluid shear stress, cyclic stress of the collateral arteriole poses another means of activating the endothelium and increasing collateral proliferation. Here, signal transduction runs via activator protein 1, among others [24].

The understanding of the physical processes of shear stress and its strong influence on collateral artery growth has led to the investigation of artificial models of excessive shear stress. In a femoral arteriovenous shunt animal model, where a surgical anastomosis was created between the femoral artery and vein distal to the occlusion of the femoral artery strongly lowered distal pressure (equaling venous pressure), thus increased pressure gradient, shear stress and finally collateral artery growth [19]. The blood flow restoration following femoral artery occlusion in this model exceeded 100% (of the contralateral, non-ligated side) easily, demonstrating that collateral arterial blood flow can actually surpass blood flow in the healthy circulation. To dissect the molecular mechanism of shear stress inducted stimulation of collateral artery growth a whole genome expression analysis from developed collateral arteries in the rabbit hindlimb was performed. Genome-wide profiling revealed actin-binding Rho-activating protein (ABRA) as one of the players most strongly upregulated and therefore potentially with a strong causal role in arteriogenesis. Indeed, overexpression and knockout experiments confirmed the pro-arteriogenic role of ABRA [18]. Further research on the exact molecular mediators of shear stress revealed the calcium channel TrpV4 to be induced by shear stress and its physical or pharmacologic activation to stimulate arteriogenesis [25]. Following complex signaling mechanisms that are beyond the scope of the present review gene expression and post-transcriptional modulation in the endothelial cell are altered and lead to enhanced activation and expression of nitric oxide synthases (NOS2 and NOS3), which both not only cause vasodilatation but also stimulate collateral arterial proliferation and growth [25,26].

These molecular changes lead to a local attraction and activation of peripheral blood monocytes. They are the most important cells during this process. Circulating monocytes transmigrate through the endothelium; they become activated and secrete matrix-degrading products such as matrix metalloproteinases (MMPs), leading to outward arterial remodeling. They also release other cytokines that orchestrate the process of arteriogenesis. For example, chemoattractants for further monocytes such as monocyte chemoattractant protein 1 (MCP-1), mitogenic factors leading to smooth muscle cell proliferation such as platelet-derived growth factor (PDGF) and tumor necrosis factor α (TNFα). The latter promotes collateral formation via its p55 receptor, as has been demonstrated in a knockout model in mice [27].

In addition, it has been debated whether pluripotent bone-marrow-derived stem cells homing to endothelium may give rise to formation of new vascular wall components [28]. Recruitment of these circulating progenitor cells (regulated by nitric oxide/reactive oxygen species balance) may relate to the molecular basis of collateral formation.

It is important to note that collateral arteries often regress once the shear stimulus has ceased. This process called ‘pruning’ finally yields few large caliber collateral arteries instead of a high number of small anastomoses [29].

In summary, the current understanding is that collateral growth (called arteriogenesis) happens via a remodeling process of pre-existing small collaterals (collateral remodeling). It differs from angiogenesis, the growth of new capillary vessels, which is induced by ischemia. Collateral growth, in contrast, is induced by fluid shear stress in preformed collateral vessels caused by a pressure gradient between the area proximal to a coronary stenosis and the low-pressure post-stenotic area. The shear stress on endothelial cells stimulates the production of nitric oxide and MCP-1, leading to an attraction of monocytes that play a key role in orchestrating collateral remodeling, including attraction of endothelial progenitor cells [30].

### Clinical significance of coronary collaterals

The clinical relevance has been disputed repeatedly since the anastomoses are often incapable of restoring flow to normal levels [31]. In fact, the presence of collaterals was sometimes even assumed to signify a worsening prognosis [20].

In the setting of an acute infarction, the relevance of coronary collaterals has been shown in preserving myocardial function [32], limiting infarct size [33], and positively influence post-infarct remodeling [34]. Increased collateral flow was also associated with less need for intra-aortic balloon pumping post-percutaneous coronary intervention (PCI) and better myocardial blush grade [35]. The presence of collaterals also appears to reduce mortality in patients, primarily due to a lower frequency of cardiogenic shock [36]. Such observations support the view that collateral flow is a modifying factor, capable of alleviating the deleterious effects of atherosclerosis on cardiovascular morbidity and mortality.

**Figure 3:**
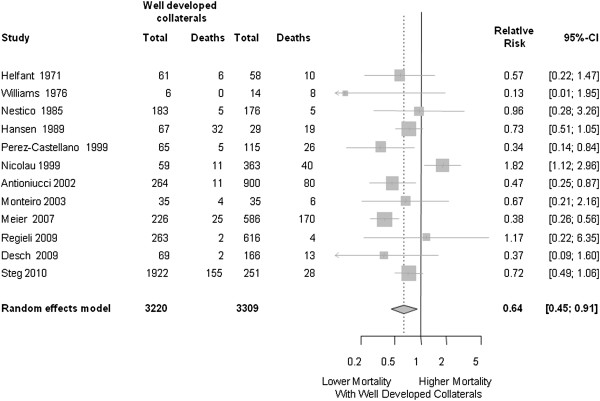
**Forest plot illustrating the results of a meta-analysis of all studies that have assessed the association between the degree of collateralization and mortality**[13]**.** 95% CI, 95% confidence interval; CCC, coronary collateral circulation; RR, relative risk.

To date, 12 studies have investigated the effect of collaterals on mortality. The first of these studies was published in 1971 in the *New England Journal of Medicine*[37]. Only three of these trials demonstrated a clear benefit for collaterals. This inconsistency did not actually help to resolve the dispute [2]. The inconsistency is partially explainable by the method of collateral assessment used in most of the studies; collaterals were ‘qualified’ visually during the coronary angiography [38]. This represents a rather crude approach. Intracoronary flow or pressure-based methods (collateral flow index) using a pressure or Doppler sensor tipped guide wire are more accurate [3]. The relevance of the collateral circulation in case of a chronic total occlusion of a coronary artery with normal left ventricular function is fairly obvious. There are even extreme examples of patients with left main artery occlusion or three-vessel occlusion with only mild symptoms [39]. Beside this anecdotal evidence, a pooled analysis of the above mentioned 12 studies (including 6,529 patients) clearly showed that, overall, well developed collaterals are associated with a reduced mortality [40]. On average, the mortality was reduced by about 35% (Figure 3).

Even though collaterals seem to have a protective effect balancing all the available evidence, they have been found to be associated with a higher risk for restenosis. A meta-analysis of 7 studies recruiting a total of 1,425 patients showed that patients with good collateralization have a 40% higher risk of restenosis as compared with patients with poor collateralization [41]. However, whether this association is causal or whether collaterals just present a risk marker is unclear. It could be an indication for the function of collaterals that prevent sufficient flow through the stented vessel. Potentially, collaterals would have been able to supply the subtended myocardium alone in these situations, rendering stenting unnecessary. Regardless, collaterals seem to be a useful and easily available marker on an individual patient level for the clinical decision-making process. In patients with better developed collaterals, cardiologists should try to reduce the risk of restenosis by using drug-eluting instead of bare-metal stents, or by prescribing cilostazol [42,43].

### Therapeutic potential

Multiple strategies to enhance collateral function have been tested (Table 1). The important role of shear stress and of monocytes have both been used as targets for the therapeutic induction of collaterals. Granulocyte-macrophage colony-stimulating factor (GM-CSF) and granulocyte colony-stimulating factor (G-CSF) are growth factors that increase monocyte numbers and they have both shown to improve collateral function [44-46]. Their mechanism of action is probably via their effect on the number but also on the gene expression profile of monocytes, a further mechanism is the release of endothelial progenitor cells from the bone marrow [46]. Another therapeutic option is to increase shear stress via external counterpulsation [47] or via physical exercise [48]; both strategies have demonstrated an effect on collateral function. External counterpulsation (ECP) can be regarded as a simulation of physical exercise in that it increases shear forces on endothelial cells. It has repeatedly been shown to reduce symptoms in patients with angina pectoris but the mechanism of action has remained unknown for years. The first controlled trial in a group of patients with coronary artery disease undergoing a 30-h program of high-pressure ECP (300 mmHg) and in a group undergoing sham ECP at 80 mmHg inflation pressure has demonstrated a relevant improvement of the collateral function (CFI) between baseline and follow-up at 4 weeks [47].

Another promising means to increase collateral artery growth is heart rate reduction using ivabradine. Bradycardia is known to be associated with better collateralization (Table 1), probably because, due to prolongation of the diastole, the lower heart rate increases the endothelial shear stress. Experimental studies indicated a benefit of ivabradine on collateral growth [49]. A clinical study to test this concept in human is currently underway (clinicaltrials.gov identifier NCT01039389); Table 2.

**Table 2:** Factors that have been tested to improve collateral circulation

**Method (references)**	**Positive effect**^**a**^	**Application**	**Remarks**
Exercise [50], [51]	Yes		No randomized data, increase in CFI.
External counterpulsation [47]	Yes		One randomized controlled trial (RCT), observational studies, increase in CFI, improvement of angina symptoms
GM-CSF [45,52]	Yes	Intracoronary, subcutaneous	Two small RCTs (n = 21, n = 12). Stopped early because of potential plaque destabilization. Increase in CFI.
G-CSF [44]	Yes	Subcutaneous	One small randomized trial (n = 52). Increase in CFI.
Dipyridamole [53]	Yes		Very small trial (n = 30); angiographic collateral assessment, which is not very accurate
VEGF [54]	No	Intracoronary	No difference in angina symptoms or exercise tolerance
FGF4 (adenovirus) [55]	No	Intracoronary	No change in exercise tolerance

^a^Based on a random effects meta-analysis model if more than one study.

*CFI*, collateral flow index; *FGF4*, fibroblast growth factor 4; *G-CSF*, granulocyte colony-stimulating factor; *GM-CSF*, granulocyte-macrophage colony-stimulating factor (GM-CSF); *VEGF*, vascular endothelial growth factor.

## Conclusions

Coronary collateral arteries serve as conduits that bridge severe stenosis or connect a territory supplied by one epicardial artery with that supplied by another. They can be recruited if required. While coronary collaterals provide substantial blood flow to the resting heart, they are often insufficient during increased myocardial oxygen demand (for example, exercise). Collateral arteries can reduce infarct size, the risk for post-infarct complications and they can also reduce mortality. Therefore, we have a considerable interest in developing methods to stimulate collateral growth. Besides known triggers of tangential shear stress and the presence of bone-marrow-derived mononuclear cells for collateral growth, first clinical proof-of-concept trials have demonstrated that collateral growth can be promoted therapeutically by physical exercise, external counterpulsation and certain growth factors and cytokines (G-CSF and GM-CSF).

## Competing interests

The authors declare that they have no competing interests with regard to this paper.

## Authors’ contributions

All authors made substantial contributions to this review article. PM, SHS and CS were involved in drafting the manuscript. AJL, AT, BP revised the manuscript critically for important intellectual content. All authors have given final approval of the version to be published.

## Authors’ information

PM is an interventional Cardiologist at University College London Hospitals UCLH, London, and part of the Yale-UCL Cardiovascular Research collaborative (http://www.drpascalmeier.com). SHS is an academic cardiologist at the University of Saarland, Germany, with a research interest in translational research in arteriogenesis/ coronary collaterals. BP is Professor Emeritus at the University of Michigan and was among the first researchers describing the existence of the coronary collateral circulation in the journal *Circulation* in 1959 [1]. AJL is a cardiologist and an Associate Professor at Yale University and a renowned expert in clinical research in the field of invasive cardiology and leading the Yale-UCL Cardiovascular Research collaborative. AT is a Professor for Cardiology and works as an interventional cardiologist in one of the highest volume cardiovascular centers in London, the London Chest Hospital. He is Editor-in-chief of the journal *Heart*. CS is a Professor of Cardiology and co-director of Cardiology at the University Hospital Bern, Switzerland. He has published widely on the topic of the coronary collateral circulation and is director of the yearly international coronary collateral symposium in Sils-Maria, Switzerland.

## Pre-publication history

The pre-publication history for this paper can be accessed here:

http://www.biomedcentral.com/1741-7015/11/143/prepub
